# Causality between thyroid disease and psoriasis: Bidirectional Mendelian randomization analysis

**DOI:** 10.1097/MD.0000000000043426

**Published:** 2025-09-05

**Authors:** Xianzhu Cong, Shuang Li, Jiayu Ge, Yuhang Zhu, Xuejie Qi, Fuyan Shi, Suzhen Wang

**Affiliations:** a Department of Health Statistics, School of Public Health, Shandong Second Medical University, Weifang, Shandong, China.

**Keywords:** genome-wide association studies, mendelian randomization, psoriasis, thyroid disease

## Abstract

Both psoriasis and autoimmune thyroid diseases are characterized by chronic inflammation. The previous studies indicated a potential association between psoriasis and autoimmune thyroid diseases. However, the direction and nature of these relationships remain unclear. This study aimed to investigate the bidirectional causal relationships between multiple thyroid diseases (hypothyroidism, hyperthyroidism, Hashimoto thyroiditis, and Graves disease (GD)) and psoriasis (PsO) as well as its subtypes (psoriasis vulgaris (PV) and psoriatic arthritis (PsA)) through a two-sample Mendelian randomization (MR) approach. A bidirectional, two-sample MR analysis was conducted using genome-wide association study (GWAS) summary statistics from large European populations. Independent single nucleotide polymorphisms (SNPs) associated with thyroid diseases and psoriasis were selected as instrumental variables. The MR-PRESSO method was applied in 2 rounds to identify and remove outlier SNPs. MR-Egger regression, inverse-variance weighted (IVW), and weighted median methods were employed to assess causal relationships. This study provides genetic evidence of a suggestive association between hypothyroidism, the most common form of thyroid disease, and an increased risk of developing PsO (odds ratio (OR) = 1.059, *P* = .038). Additionally, genetic predisposition to hypothyroidism was significantly associated with its subtype, PsA (OR = 1.184, *P* = 2.40 × 10^−5^). In the inverse MR analyses, PV, the primary subtype of PsO, was suggestively associated with an increased risk of GD (OR = 1.113, *P* = .002) and hyperthyroidism (OR = 1.053, *P* = .040). However, it was associated with a decreased risk of hypothyroidism (OR = 0.977, *P* = .025). This study presents evidence supporting bidirectional causal relationships between thyroid diseases and psoriasis. These findings offer new insights into shared inflammatory pathways that may underlie these comorbidities.

## 1. Introduction

Thyroid disease is very common worldwide, with about 20 million people in the United States and 200 million in China affected.^[1–3]^ It disrupts hormone production, particularly thyroxine (T4) and triiodothyronine (T3).^[4]^ Autoimmune thyroid diseases, such as Graves disease (GD) and Hashimoto thyroiditis (HT), are common causes.^[5]^ GD often leads to hyperthyroidism, characterized by excessive hormone secretion, which manifests as weight loss, tachycardia, and anxiety.^[6]^ In contrast, HT frequently results in hypothyroidism, leading to fatigue, weight gain, and depression.^[7]^ Both conditions arise from the immune system attacking the thyroid, affecting its function.

Psoriasis (PsO) is a chronic inflammatory disease characterized by immune-mediated, polygenic inheritance and triggered by multiple environmental factors.^[8]^ It is categorized into 4 main types: psoriasis vulgaris (PV), psoriatic arthritis (PsA), generalized pustular psoriasis, and erythrodermic psoriasis. PV is the most common type, followed by PsA.^[9]^ The World Health Organization classifies psoriasis as a major non-communicable disease that affects over 60 million adults and children globally.^[10]^ Previous studies have demonstrated a higher incidence of psoriasis in Caucasians.^[11]^ The prevalence in East Asia is 0.14%, while in Western and Central Europe, the prevalence rates are 1.92% and 1.83%, respectively. Psoriasis significantly impairs the quality of life of patients, placing them under considerable stress and particularly affecting their psychosocial well-being.^[12,13]^

As an autoimmune disease, psoriasis is strongly associated with GD and HT.^[14–16]^ Namiki et al proposed a possible association between psoriasis and thyroid dysfunction.^[17]^ Additionally, Kiguradze et al demonstrated a significant association between psoriasis and HT (*P* < .05, OR = 2.49) in a cross-sectional study.^[18]^ Similarly, Cira et al described associations between psoriasis, its subtypes, and thyroid disorders.^[19]^ This review highlights the challenges in establishing a clear causal relationship between psoriasis and thyroid diseases. Most data on their association are derived from observational studies. However, due to the inherent limitations of observational research, including lack of randomization and potential reverse causality, the inconsistent findings across multiple studies hinder definitive conclusions. These limitations raise concerns regarding the reliability of causal inferences in this context. Therefore, it is essential to employ research methods that control for confounding factors and mitigate reverse causality to further explore the causal relationship between psoriasis and thyroid disease. This will offer novel insights into the clinical management and prevention of psoriasis and thyroid diseases.

Mendelian randomization (MR) employs single nucleotide polymorphisms (SNPs) as instrumental variables (IVs) to establish a nature group and offer a robust method for addressing potential confounders and biases inherent in observational studies so as to obtain a reliable causal relationship at the genetic level, and also providing reverse causality.^[20,21]^ According to Mendel principle of independent assessment, random segregation of alleles can achieve a uniform distribution of unmeasured confounding factors between 2 cohorts, thereby enhancing the accuracy of inferences regarding causal relationships between psoriasis and thyroid disease.^[22]^ Bidirectional MR studies play important roles in elucidating the direction of causal relationships for genetic data.^[23,24]^ This study aims to examine the strength of association and direction of causality between psoriasis and thyroid disease through a bidirectional, two-sample MR approach, utilizing genome-wide association studies (GWAS) summary statistics.

## 2. Materials and methods

### 2.1. Study design

**Figure 1.**
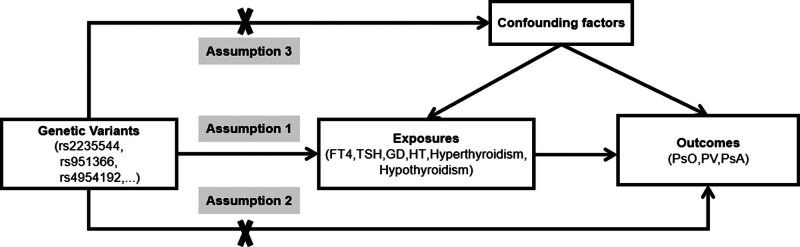
Unidirectional MR from thyroid disease to psoriasis and its subtypes. Assumption 1: There is a strong correlation between the instrumental variable and the exposure; Assumption 2: The instrumental variable is not correlated with confounding factors that influence the outcome; Assumption 3: The instrumental variable solely impacts the outcome through exposure factors, not other means. GD = Graves disease, HT = Hashimoto thyroiditis, MR = Mendelian randomization, PsA = psoriatic arthritis, PsO = psoriasis, PV = psoriasis vulgaris, TSH = thyroid stimulating hormone.

**Figure 2.**
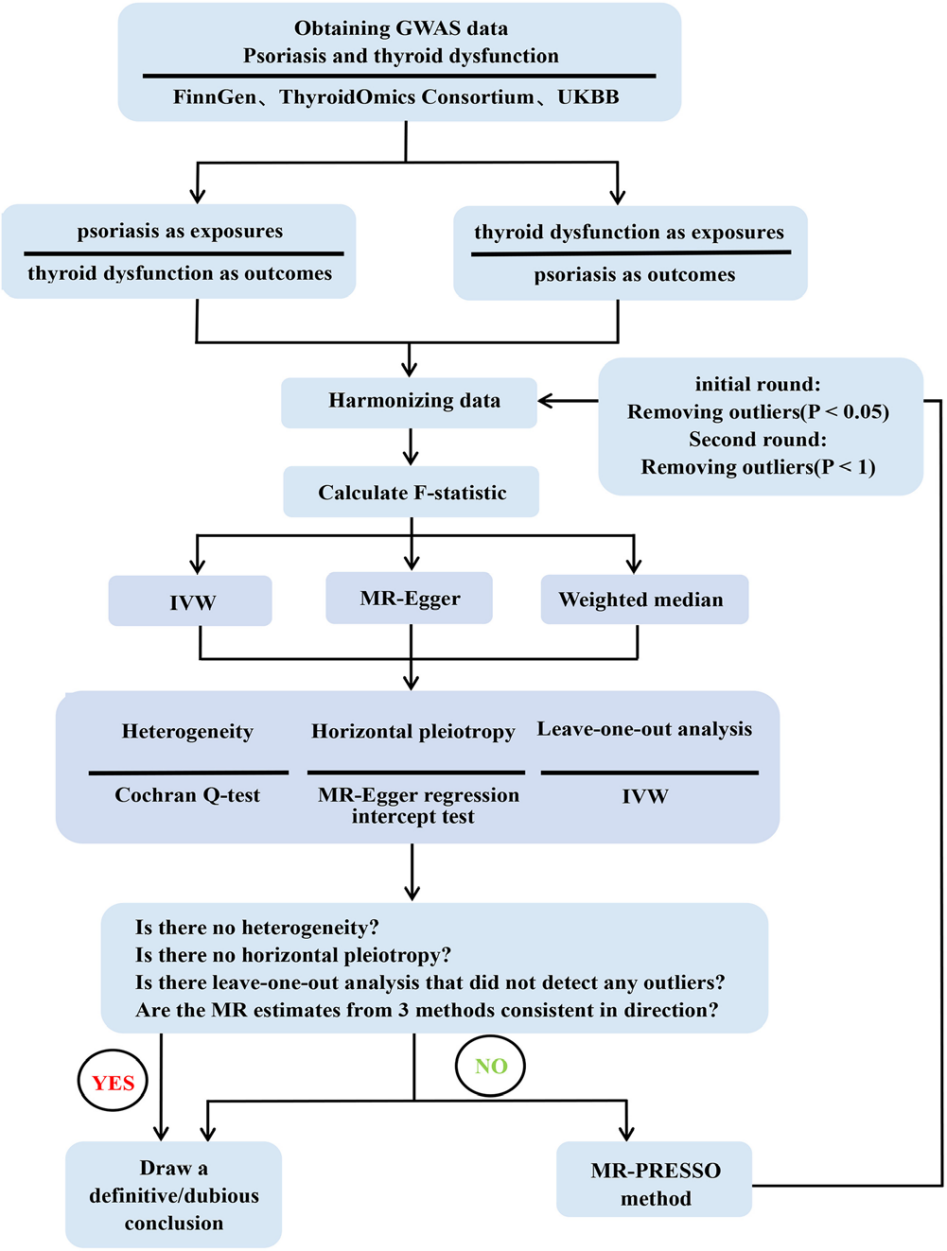
Flow chart for bidirectional MR analysis design. The black arrow indicates the sequence of the process and displays the analysis process step by step. GWAS = genome-wide association studies, IVW = inverse-variance weighted method, MR = Mendelian randomization.

For this study, we utilized GWAS data from individuals of European ancestry. All individual studies obtained approval from their respective local institutional review boards and ethics committees, with written informed consent obtained from all participants. Consent for minors was obtained from parents or legal guardians. Since this study only involved secondary analyses of publicly available summary statistics, additional ethical approval or consent from participants was not required. To ensure the robustness of the results, MR analysis must satisfy the 3 fundamental assumptions depicted in Figure 1.^[25]^ A study design framework is illustrated in Figure 2 to represent the structure of our study.

### 2.2. Data source

This study obtained summary data from GWAS studies on PsO, PV, and PsA through the FinnGen study.^[26]^ Summary data on GD, HT, hyperthyroidism, hypothyroidism, FT4, and TSH were acquired from the IEU OpenGWAS^[27]^ project and the ThyroidOmics Consortium.^[28]^ When there are many GWAS summary datasets available for a certain project, the criteria for choosing the best GWAS summary data are as follows: The study was carried out on individuals of European descent; The GWAS data used is independent of the study outcomes; Complete GWAS summary data was accessible; There were a larger number of cases available for analysis; There were a greater number of test SNPs available for analysis. Table 1 shows a summary of the data, including data sources and sample sizes.

**Table 1:** Summary information of GWAS data.

Exposure or outcome	GWAS_PMID*	Sample size	Web source†
Hyperthyroidism	34594039	ncase: 3557 ncontrol: 456,942	MR-base: ebi-a-GCST90018860
Hypothyroidism	34594039	ncase: 30,155 ncontrol: 379,986	MR-base: ebi-a-GCST90018862
Graves disease	34594039	ncase: 1678 ncontrol: 456,942	MR-base: ebi-a-GCST90018847
Hashimoto thyroiditis	34594039	ncase: 15,654 ncontrol: 379,986	MR-base: ebi-a-GCST90018855
FT4	30367059	Total number: 72,167	ThyroidOmics Consortium
TSH	30367059	Total number: 72,167	ThyroidOmics Consortium
Psoriasis	FinnGen	ncase: 9267 ncontrol: 364,071	FinnGen-R9
Psoriasis vulgaris	FinnGen	ncase: 679 ncontrol: 364,071	FinnGen-R9
psoriatic arthritis	FinnGen	ncase: 3186 ncontrol: 240,862	FinnGen-R9

GWAS = genome-wide association studies, MR = Mendelian randomization.

* GWAS_PMID is the PubMed ID for GWAS of outcomes. FinnGen means the GWAS was derived from the FinnGen research project, respectively.

† MR-base: https://gwas.mrcieu.ac.uk/; FinnGen-R9: https://www.finngen.fi/en; ThyroidOmics Consortium: https://www.thyroidomics.com.

### 2.3. Selection of IVs

We employed the 1000 Genomes European Reference Panel and applied the PLINK clumping method to evaluate linkage disequilibrium (LD) among SNPs, selecting independent SNPs without LD as IVs. The selection criteria for SNPs were as follows: genome-wide significance, defined as *P* < 5 × 10^−8^, was used to identify relevant SNPs from GWAS data; to minimize the impact of LD, SNPs were required to have an *r*² < 0.001 and a genetic distance of 10,000 kb; when specific exposure data for a SNP was unavailable, proxy SNPs were not considered; SNPs were required to show no significant association with potential confounding factors, as verified using the PhenoScanner database (https://www.phenoscanner.medschl.cam.ac.uk/phenoscanner); SNP harmonization was performed to ensure correct allelic alignment, excluding palindromic SNPs; and to address potential weak IVs, an F-statistic >10 was applied to assess the strength of each IV. The F-statistic was derived using the formula $F=\frac{R^{2}\left(N-2\right)}{\left(1-R^{2}\right)}$, where $R^{2}$ reflects the proportion of exposure variance explained by the IV. $R^{2}$ was obtained using $2\times \beta^{2}\times EAF\times \left(1-EAF\right)$. The variable N shows the number of people exposed to the GWAS data, β is the regression coefficient for the mean IV, and EAF is the effect of allele frequency.^[29,30]^ The final retained SNPs were used as IVs for MR analysis.

### 2.4. Mendelian randomization

Three methods with distinct statistical assumptions, namely inverse-variance weighted (IVW), MR-Egger regression, and weighted median, were utilized for the MR analyses. These methods have been widely applied, and our conclusions are primarily based on the IVW method. For datasets containing 3 or fewer SNPs as IVs, the IVW fixed-effects model analysis was conducted. For datasets containing more than 3 SNPs as IVs, the IVW analysis was performed using the multiplicative random-effects model.^[31]^ The MR-Egger and weighted median methods were applied to adjust the IVW estimates when there were potentially erroneous IVs. The MR-Egger method can handle horizontal pleiotropy but is sensitive to outliers and violations of the Instrument Strength Independent of Direct Effect (InSIDE) assumption, whereas the weighted median is more robust to the influence of outliers.^[25,32,33]^

A preliminary MR analysis was performed using the Cochran *Q* test to assess SNP heterogeneity, and no evidence of heterogeneity was found. The MR-Egger regression intercept test indicated no evidence of directional pleiotropy, and the MR-PRESSO analysis did not detect any outliers. Based on the fulfillment of these 3 conditions, the effect directions estimated by the 3 MR methods were consistent, allowing us to derive reliable conclusions. As depicted in Figure 2, when the conditions for drawing reliable conclusions were not met, 2 rounds of MR-PRESSO analysis were carried out to reduce the effect of SNP outliers while retaining the maximum number of SNPs.^[34]^ During the initial round of MR-PRESSO analysis, outliers were identified based on a significance threshold of *P* < .05. In the second round, a more lenient threshold of *P* < 1 was applied. After each round, outliers were removed, and the MR analysis was repeated using the remaining SNPs as IVs. If more than half of the instrumental SNPs were outliers, MR analysis was discontinued. Similarly, if fewer than 3 SNPs remained as IVs after removing outliers, MR analysis was not repeated. After 2 rounds of the MR-PRESSO analysis, if horizontal pleiotropy, outliers, or inconsistent directions remained, only dubious conclusions could be drawn. Conversely, if these problems did not persist, reliable conclusions were reached. Reverse MR analysis, after swapping exposure and outcome, use the same analysis process.

We conducted a power calculation to determine the ability of our MR analysis to identify associations between genetically predicted exposures and outcomes. The variance in exposures explained by the IVs was estimated using $R^{2}$. The mRnd online tool (http://cnsgenomics.com/shiny/mRnd/) was utilized to perform the power analysis (Table S1, Supplemental Digital Content, https://links.lww.com/MD/P729). Assess the cause effect connection between exposure and outcomes by computing the odds ratio (OR) and its 95% confidence interval (CI). To address the issue of multiple testing, Bonferroni-corrected significance levels were employed (*P* < .05/number of tests). The one-way MR comparison in this study was conducted 18 times, so the significance level threshold for this study is 2.8 × 10^−3^. The statistical analyses were conducted using the TwoSampleMR^[27]^ and MR-PRESSO^[35]^ packages in the R program (version 4.3.1).

### 2.5. Sensitivity analyses

To assess heterogeneity and horizontal pleiotropy among SNPs, a series of sensitivity analyses were conducted utilizing the Cochran *Q* test and the MR-Egger regression intercept test. This study conducted a leave-one-out analysis utilizing the IVW approach to examine if the inclusion of SNPs had a statistically significant impact on the outcomes. Funnel plots assess potential bias and pleiotropy by checking the symmetry of effect estimates, while forest plots display and compare the effects of individual genetic instruments and overall results.

## 3. Results

### 3.1. Statistics for IVs

We employed 15, 27, 13, 75, 24, 45, 33, 6, and 14 independent SNPs without LD as IVs for HT, GD, hyperthyroidism, hypothyroidism, FT4, TSH, PsO, PV, and PsA, respectively (Table S2, Supplemental Digital Content, https://links.lww.com/MD/P730). Depending on the each exposure-outcome pairs, palindromic SNPs were eliminated, and several outlier SNPs were identified and excluded using the MR-PRESSO method. The number of SNPs used as IVs ranged from 3 to 62. $R^{2}$ ranging from 3.298% to 66.265% (Table S1, Supplemental Digital Content, https://links.lww.com/MD/P729). All SNPs utilized as IVs exhibited F-statistic values >10, indicating the absence of weak IVs (Table S3, Supplemental Digital Content, https://links.lww.com/MD/P731).

### 3.2. Causal effects of thyroid diseases on psoriasis and its subtypes

**Figure 3.**
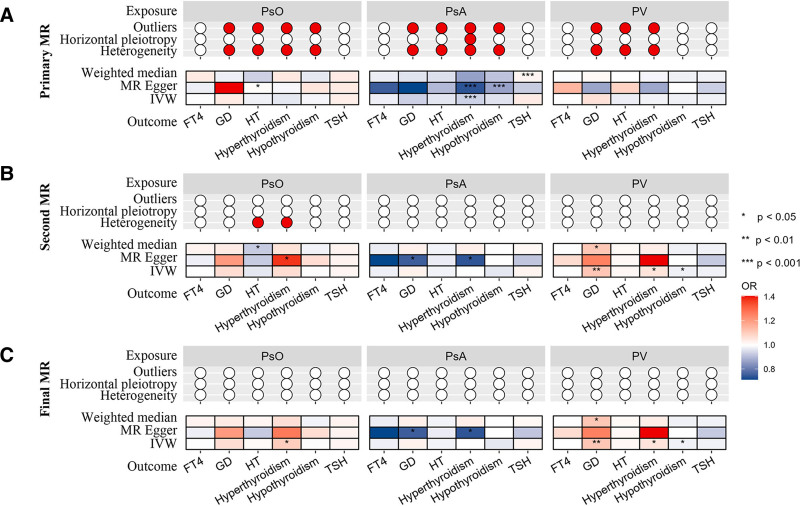
Association estimates of autoimmune thyroid diseases with psoriasis and its subtypes in MR analysis. (A) primary MR analysis; (B) second MR analysis with the exclusion of outlying genetic variants (*P* < .05) detected in MR-PRESSO analysis; (C) final MR analysis with the exclusion of outlying genetic variants (*P* < 1) detected in MR-PRESSO analysis. Red dots denote the presence of outliers, horizontal pleiotropy, or heterogeneity. GD = Graves disease, HT = Hashimoto thyroiditis, MR = Mendelian randomization, OR = odds ratio, PsA = psoriatic arthritis, PsO = psoriasis, PV = psoriasis vulgaris, TSH = thyroid stimulating hormone. *** *P* < .001; ** *P* < .01; * *P* < .05.

In the primary MR analysis of psoriasis and its subtypes, Cochran *Q* test detected heterogeneity in SNPs among 10 exposure-outcome pairs, whereas MR-Egger regression did not detect horizontal pleiotropy in any IVs (Fig. 3). After 2 rounds of MR-PRESSO to detect and remove outliers, the final MR analysis showed no heterogeneity and no horizontal pleiotropy. The scatter plots, funnel plots, forest plots, and leave-one-out analysis plots are available in Figures S1 to S4 (Supplemental Digital Content, https://links.lww.com/MD/P728).

Reliable conclusions can be drawn when estimates across the 3 MR methods are consistent, showing no heterogeneity or horizontal pleiotropy. Conversely, conclusions become uncertain when estimates are inconsistent or when heterogeneity or horizontal pleiotropy is present. In the final MR analysis, 2 reliable conclusions were reached. A significant causal relationship was identified between hypothyroidism and PsA (OR = 1.184, 95% CI: 1.095–1.280, *P* = 2.40 × 10^−5^; *P* < 2.8 × 10^−3^), and a suggestive causal relationship between hypothyroidism and PsO (OR = 1.059, 95% CI: 1.003–1.118, *P* = .038, 2.8 × 10^−3^ <* P* < .05) was observed in the IVW analysis. Additionally, inconsistent OR values across the 3 methods led to 2 dubious conclusions. A suggestive causal relationship between HT and PsO (OR = 1.076, 95% CI: 1.003–1.155, *P* = .040, 2.8 × 10^−3^ < *P* < .05) and between HT and PsA (OR = 1.170, 95% CI: 1.035–1.324, *P* = .012, 2.8 × 10^−3^ < *P* < .05) in the IVW analysis. No causal relationship was detected for the remaining exposure-outcome pairs. Further details of the MR analyses can be found in Figure 3, Table 2, Table S4 (Supplemental Digital Content, https://links.lww.com/MD/P732), and Table S5 (Supplemental Digital Content, https://links.lww.com/MD/P733).

**Table 2:** MR analysis results with statistical significance.

Exposure	Outcome	Method	*P* value	OR (95% CI)
HT	PsO	IVW*	.04	1.076 (1.003–1.155)
HT	PsO	MR Egger*	.797	0.975 (0.813–1.169)
HT	PsO	Weighted median*	.159	1.073 (0.973–1.183)
HT	PsA	IVW*	.012	1.170 (1.035,1.324)
HT	PsA	MR Egger*	.728	0.942 (0.681–1.304)
HT	PsA	Weighted median*	.001	1.278 (1.105–1.479)
Hypothyroidism	PsO	IVW†	.038	1.059 (1.003–1.118)
Hypothyroidism	PsO	MR Egger†	.809	1.016 (0.896–1.151)
Hypothyroidism	PsO	Weighted median*	.065	1.072 (0.996–1.155)
Hypothyroidism	PsA	IVW†	2.40 × 10^−5^	1.184 (1.095–1.280)
Hypothyroidism	PsA	MR Egger†	.141	1.147 (0.958–1.373)
Hypothyroidism	PsA	Weighted median*	.001	1.234 (1.090–1.398)
PV	Hypothyroidism	IVW‡	.025	0.977 (0.957–0.997)
PV	Hypothyroidism	MR Egger‡	.976	0.999 (0.951–1.050)
PV	Hypothyroidism	Weighted median*	.059	0.976 (0.952–1.001)
PV	Hyperthyroidism	IVW*	.04	1.053 (1.002–1.106)
PV	Hyperthyroidism	MR Egger*	.213	1.404 (0.921–2.140)
PV	Hyperthyroidism	Weighted median*	.41	1.033 (0.956–1.116)
PV	GD	IVW*	.002	1.113 (1.040–1.191)
PV	GD	MR Egger*	.505	1.251 (0.700–2.235)
PV	GD	Weighted median*	.015	1.112 (1.021–1.212)
PsO	Hyperthyroidism	IVW†	.032	1.100 (1.008–1.201)
PsO	Hyperthyroidism	MR Egger†	.065	1.264 (0.998–1.599)
PsO	Hyperthyroidism	Weighted median*	.256	1.064 (0.956–1.184)

GD = Graves disease, HT = Hashimoto thyroiditis, IVW = inverse-variance weighting, MR = Mendelian randomization, OR = odds ratio, PsA = psoriatic arthritis, PsO = psoriasis, PV = psoriasis vulgaris, TSH = thyroid stimulating hormone.

* Second MR analysis results with statistical significance.

† Final MR analysis results with statistical significance.

‡ Primary MR analysis results with statistical significance.

### 3.3. Causal effects of psoriasis and its subtypes on thyroid diseases

**Figure 4.**
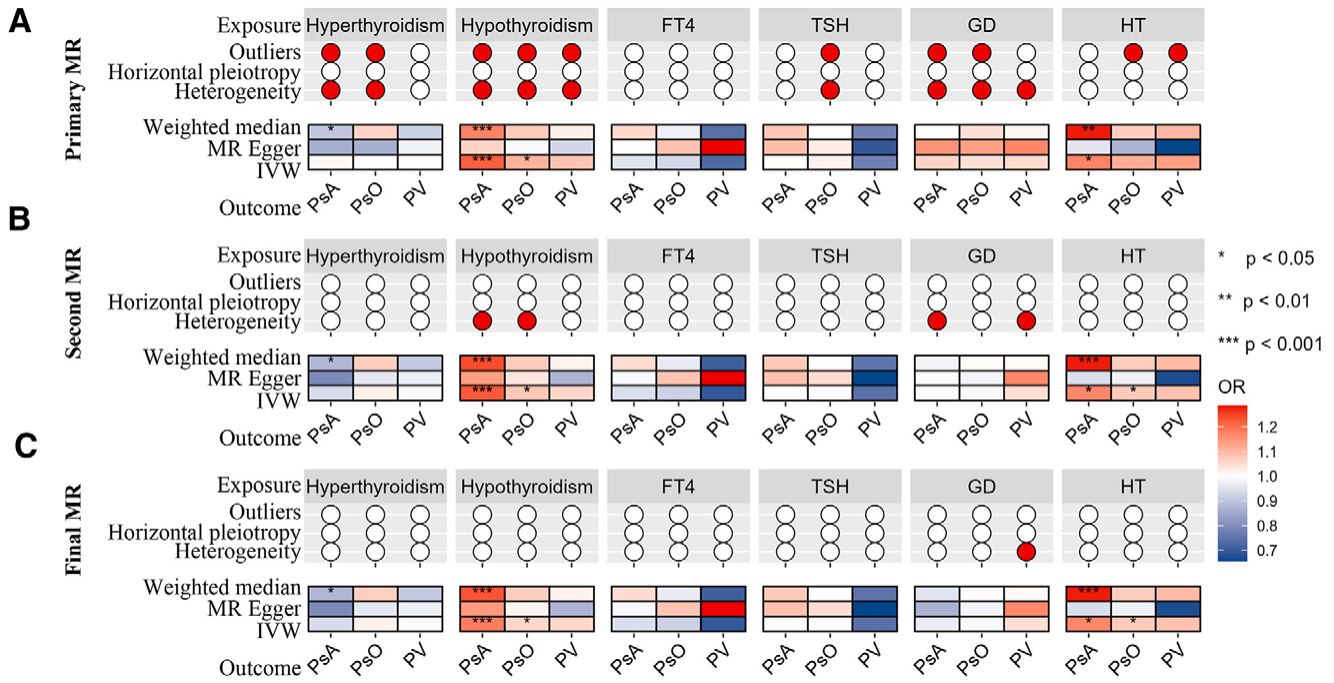
Association estimates of psoriasis and its subtypes with autoimmune thyroid diseases in MR analysis. (A) primary MR analysis; (B) second MR analysis with the exclusion of outlying genetic variants (*P *< .05) detected in MR-PRESSO analysis; (C), final MR analysis with the exclusion of outlying genetic variants (*P* < 1) detected in MR-PRESSO analysis. Red dots denote the presence of outliers, horizontal pleiotropy, or heterogeneity. GD = Graves disease, HT = Hashimoto thyroiditis, MR = Mendelian randomization, OR = odds ratio, PsA = psoriatic arthritis, PsO = psoriasis, PV = psoriasis vulgaris, TSH = thyroid stimulating hormone. *** *P* < .001; ** *P* < .01; * *P* < .05.

In the primary MR analysis of thyroid diseases, Cochran *Q* test detected heterogeneity among SNPs in 11 exposure-outcome IVs, while MR-Egger regression revealed horizontal pleiotropy between PsA and SNPs associated with hyperthyroidism (Fig. 4). After 2 rounds of MR-PRESSO to detect and remove outliers, the final MR analysis showed no heterogeneity and no horizontal pleiotropy. The scatter plots, funnel plots, forest plots, and leave-one-out analysis plots are available in Figures S1 to S4 (Supplemental Digital Content, https://links.lww.com/MD/P728).

Four reliable conclusions were identified in the final MR analysis. A suggestive causal relationship was identified between PV and hypothyroidism (OR = 0.977, 95% CI: 0.957–0.997, *P *= .025, 2.8 × 10^−3^ < *P *< .05), and another suggestive causal relationship was found between PV and GD (OR = 1.113, 95% CI: 1.040–1.191, *P *= .002, 2.8 × 10^−3^ < *P *< .05). Additionally, a suggestive causal relationship was observed between PV and hyperthyroidism (OR = 1.053, 95% CI: 1.002–1.106, *P *= .040, 2.8 × 10^−3^ < *P *< .05). Similarly, PsO showed a suggestive causal relationship with hyperthyroidism (OR = 1.100, 95% CI: 1.008–1.201, *P* = .032, 2.8 × 10^−3^ < *P* < .05). No causal relationship was detected for the remaining exposure-outcome pairs. Further details of the MR analyses can be found in Figure 4, Table 2, Table S4 (Supplemental Digital Content, https://links.lww.com/MD/P732), and Table S5 (Supplemental Digital Content, https://links.lww.com/MD/P733).

## 4. Discussion

### 4.1. Principal findings

This study employed a bidirectional, two-sample MR method to investigate the causal associations between psoriasis, its subtypes, and thyroid disease. The MR study indicated a statistically significant causal association between PsO and an increased susceptibility to hyperthyroidism. There was a statistically significant causal association between PV and an increased susceptibility to GD and hyperthyroidism. There was a causal association between PV and a reduced risk of hypothyroidism. Additionally, the results of the MR analysis indicated a strong causal association between hypothyroidism and an increased likelihood of developing PsO. There was a causal association between hypothyroidism and an increased risk of PsA. A weak causal association was observed between HT and an increased likelihood of both PsO and PsA, which warranted further investigation. In addition, bidirectional MR investigations did not identify other causal associations between illnesses.

### 4.2. Comparison with other studies

In our MR study examining the causal relationship between psoriasis and its subtypes and thyroid disease, we identified a positive causal effect of plaque PsO on hyperthyroidism. This association was consistently validated across all 3 MR methods, highlighting the robustness and reliability of the findings. Consistent with previous cohort studies, the results indicate an increased likelihood of hyperthyroidism in individuals with PsO.^[36]^ Considering that PV is the predominant type of PsO and GD is the leading cause of hyperthyroidism, the observed positive causal effect of PV on hyperthyroidism, akin to its effect on GD, aligns with earlier observational studies.^[6,19,36,37]^ These findings further suggest that PsO may causally increase the risk of hyperthyroidism. In contrast to prior studies, the present study concludes that PV is associated with a increased risk of developing hyperthyroidism. Most previous studies found no significant difference in the incidence of hyperthyroidism between psoriasis patients and the normal population. The differing conclusions in this study may stem from the stringent criteria applied in GWAS data to select patients with PV, thereby excluding the impact of other psoriasis subtypes or confounding factors.

In the MR analysis examining the causal impact of thyroid disease on psoriasis and its subtypes, the findings suggest that hypothyroidism elevates the risk of developing PsO and PsA. The most robust result is observed for the association between hypothyroidism and PsA, a conclusion substantiated across IVW methods after Bonferroni correction. Earlier studies predominantly indicated an increased prevalence of autoimmune thyroid disorders or thyroid dysfunction in psoriasis patients (OR > 1).^[38,39]^ Similarly, the current analysis reveals that hypothyroidism, as a risk factor, heightens the likelihood of developing PsO and PsA. This may be attributed to genetic factors acting as IVs that mitigate reverse causality. Further exploration could investigate whether, in the real world, the prevalence of PsO and PsA is higher in individuals with hypothyroidism compared to the general population, shedding light on the role of thyroid function in psoriasis patients.

Our analysis revealed that hypothyroidism, as a risk factor, is associated with an increased likelihood of developing plaque PsO and PsA. This association may be influenced by genetic factors acting as IVs, which reduce the impact of reverse causality. Further research could examine whether individuals with hypothyroidism have a higher prevalence of PsO and PsA compared to the general population, providing valuable insights into the role of thyroid function in psoriasis. The causal effect of HT on the increased risk of PsO and PsA was confirmed exclusively by the IVW-MRE method. Consistent with previous studies, our findings align with reports of a heightened risk of autoimmune thyroid disorders, particularly HT, among patients with PsO and PsA, highlighting a notable overlap in the prevalence of these conditions.^[40,41]^ However, there have been no studies examining PsO and PsA risk in patients with HT to date.

In this MR analysis, HT was found to be a risk factor for PsO and PsA, which may explain the high prevalence of HT in patients with PsO and PsA. As mentioned above, the results of MR may reduce the effect of reverse causation and indicate a true causal relationship.

### 4.3. Biological mechanisms

The association between psoriasis, its subtypes, and thyroid diseases is complex and not fully elucidated. However, emerging evidence underscores a close relationship driven by shared inflammatory pathways. Psoriasis is predominantly mediated by pro-inflammatory cytokines, including TNF-α, IL-17, IL-22, and IFN-γ, secreted by activated T cells. Central to its pathogenesis is the TNF-IL-23-IL-17 axis, which also plays a pivotal role in autoimmune thyroid diseases such as GD and HT. In this cascade, TNF-α acts as a critical regulator of the IL-23/IL-17 axis by promoting Th17 cell differentiation and proliferation. Activated Th17 cells secrete IL-17, which directly stimulates keratinocytes to produce various chemokines, including CXCL9 and CXCL10. Notably, these chemokines are elevated in both GD and psoriasis patients, further highlighting the interconnected inflammatory mechanisms underlying these conditions.^[39,42–44]^

Research has also revealed a close association between Th17 cells, IL-17, and autoimmune thyroid disorders. For instance, a study by Torimoto et al suggested that elevated Th17 cells and IL-17 levels in GD and HT patients indicate a critical role for the TNF-IL-23-IL-17 axis in autoimmune thyroid disease.^[45]^ In addition to shared cytokine pathways, the connection between psoriasis and thyroid disease is also reflected in the dysregulation of the NF-κB pathway, a downstream effector of TNF-α signaling.^[46]^ NF-κB is a transcription factor that promotes the expression of pro-inflammatory genes and is a common feature of both psoriasis and thyroid autoimmunity, further suggesting that these conditions share molecular mechanisms.^[47]^ Subtypes of psoriasis, such as plaque PsO and PsA, exhibit distinct inflammatory profiles. Plaque PsO is characterized by elevated levels of IL-17 and TNF-α, whereas PsA is associated not only with increased IL-17 levels but also with additional pro-inflammatory cytokines, such as IL-6 and IL-1β. Despite these differences, both conditions share a common underlying mechanism driven by dysregulation of the IL-23/IL-17 axis.^[48,49]^ The systemic inflammation characteristic of PsA, driven by elevated levels of IL-17 and TNF-α, may contribute to the heightened risk of thyroid diseases in psoriasis patients. Elevated levels of IL-17 and Th17 cells observed in both GD and HT further suggest that a shared inflammatory axis may play a key role in the comorbidity between these conditions.^[50]^

Overall, the TNF-IL-23-IL-17 pathway appears to be a critical link between psoriasis, its subtypes, and thyroid diseases, offering potential therapeutic targets for managing both conditions. Further research is needed to fully elucidate these shared mechanisms, but the evidence so far underscores the importance of the TNF-IL-23-IL-17 axis in driving the inflammatory processes underlying psoriasis and autoimmune thyroid disorders.

### 4.4. Strengths and limitations of the study

This study employed a two-sample MR approach, using genetic variants as IVs. Since genetic variants are fixed at conception, this method minimizes confounding and reverse causality by reflecting the lifelong effects of risk factors. The study utilized GWAS data on psoriasis and thyroid-related diseases from large European populations across diverse databases, helping to mitigate sample overlap, reduce the risk of type I errors, and minimize bias from ethnic, lifestyle, and dietary differences.

Despite these advantages, the study has several limitations. First, the MR analysis relies on GWAS summary data from public databases. Despite rigorous screening, the sample size may still be insufficient, potentially introducing bias in identifying disease-associated SNPs. Second, the use of GWAS data from predominantly European populations may limit the generalizability of the findings to non-European racial and ethnic groups.

## 5. Conclusion

In summary, this MR study presents strong evidence supporting bidirectional causal relationships between thyroid diseases and psoriasis. These results offer new insights into the shared inflammatory pathways between thyroid diseases and psoriasis, particularly involving the TNF-IL-23-IL-17 axis. Further research is needed to confirm these findings in diverse populations and to explore the underlying biological mechanisms connecting these conditions.

## Acknowledgments

Thanks to the thousands of people who have shared their genotype information and conducted GWAS surveys and studies. In particular, we want to acknowledge the participants and investigators of the FinnGen study, the ThyroidOmics Consortium study, and the UKBB GWAS participants.

## Author contributions

**Data curation:** Xianzhu Cong.

**Formal analysis:** Fuyan Shi.

**Funding acquisition:** Suzhen Wang.

**Investigation:** Fuyan Shi.

**Methodology:** Shuang Li, Jiayu Ge, Yuhang Zhu, Xuejie Qi.

**Writing – original draft:** Xianzhu Cong.

**Writing – review & editing:** Yuhang Zhu, Fuyan Shi, Suzhen Wang.

## Supplementary Material


