# Supplementary material for: Causality between thyroid disease and psoriasis: Bidirectional Mendelian randomization analysis
Source: Medicine (Baltimore). 2025 Sep 5;104(36):e43426. doi: 10.1097/MD.0000000000043426 (PMC12419343; doi:10.1097/MD.0000000000043426)
Supplement: Supplementary file 1 [file medi-104-e43426-s001.docx]

**Table S1** Statistics for instrumental variables and removed SNPs

| exposure | outcome | nsnp | R2（%） | power（%） | Remove SNP |  |
| --- | --- | --- | --- | --- | --- | --- |
| HT | PsO | 7 | 6.497 | 46.06 | rs78765971、rs1993945、rs9357119、rs3997998、rs9271365、rs7754251、rs3184504、rs757024 | |
| HT | PsA | 11 | 12.871 | 35.54 | rs78765971、rs1993945、rs7754251、rs757024 | |
| HT | PV | 9 | 11.069 | 37.16 | rs78765971、rs1993945、rs9357119、rs3997998、rs7754251、rs757024 | |
| GD | PsO | 19 | 32.987 | 9.33 | rs9260041、rs7741597、rs7746061、rs9273410、rs7754251、rs61226717、rs11643872、rs5912815 | |
| GD | PsA | 18 | 30.263 | 7.76 | rs6679677、rs9260041、rs7741597、rs6936707、rs9273410、rs7754251、rs61226717、rs11643872、rs5912815 | |
| GD | PV | 22 | 40.558 | 40.69 | rs9273410、rs7754251、rs61226717、rs11643872、rs5912815 | |
| Hyperthyroidism | PsO | 9 | 13.943 | 11.09 | rs385863、rs1794280、rs2856821、rs28375776 | |
| Hyperthyroidism | PsA | 9 | 17.53 | 10.39 | rs6679677、rs385863、rs2856821、rs28375776 | |
| Hyperthyroidism | PV | 11 | 20.358 | 5.48 | rs385863、rs28375776 | |
| Hypothyroidism | PsO | 53 | 18.506 | 68.40 | rs78765971、rs12117927、rs11420448、rs2111485、rs307558、rs10075764、rs9264277、rs28418426、rs1065386、rs9271365、rs9273371、rs9277559、rs141232332、rs2921053、rs12379417、rs3118469、rs61877856、rs3184504、rs11406335、rs34536443、rs2412976、rs5912815 | |
| Hypothyroidism | PsA | 57 | 20.383 | 58.08 | rs78765971、rs11420448、rs2111485、rs307558、rs434294、rs9264277、rs28418426、rs1065386、rs9271365、rs9273371、rs9277559、rs141232332、rs2921053、rs61877856、rs11406335、rs34536443、rs2412976、rs5912815 | |
| Hypothyroidism | PV | 62 | 24.883 | 37.15 | rs78765971、rs11420448、rs307558、rs9264277、rs28418426、rs1065386、rs9273371、rs141232332、rs2921053、rs11406335、rs34536443、rs2412976、rs5912815 | |
| FT4 | PsO | 21 | 3.298 | 28.85 |  | |
| FT4 | PsA | 21 | 3.298 | 5.81 |  | |
| FT4 | PV | 21 | 3.298 | 88.32 |  | |
| TSH | PsO | 40 | 7.058 | 5.37 | rs2359775、rs12364、rs118039499、rs8176645、rs199461 | |
| TSH | PsA | 41 | 7.124 | 5.01 | rs2359775、rs118039499、rs8176645、rs199461 | |
| TSH | PV | 41 | 7.124 | 97.30 | rs2359775、rs118039499、rs8176645、rs199461 | |
| PsO | Hyperthyroidism | 23 | 16.48 | 67.70 | rs13210419、rs805285、rs9273060、rs3129207、rs4713637、rs2664280、rs1250566、rs7310615、rs2021511、rs34536443 | |
| PsO | Hypothyroidism | 19 | 13.581 | 7.32 | rs78456138、rs74817271、rs13210419、rs805285、rs9273060、rs3129207、rs181316459、rs60600003、rs16903065、rs2664280、rs1250566、rs7310615、rs2021511、rs34536443 | |
| PsO | FT4 | 21 | 11.39 | 3.10 | rs78456138、rs62396280、rs805285、rs9273060、rs3129207、rs181316459、rs2664280、rs7310615、rs144651842 | |
| PsO | TSH | 21 | 11.39 | 37.10 | rs78456138、rs62396280、rs805285、rs9273060、rs3129207、rs181316459、rs2664280、rs7310615、rs144651842 | |
| PsO | GD | 25 | 15.232 | 18.82 | rs1611704、rs13210419、rs805285、rs9273060、rs3129207、rs4713637、rs2664280、rs7310615 | |
| PsO | HT | 23 | 15.716 | 7.31 | rs78456138、rs13210419、rs805285、rs9273060、rs3129207、rs9346778、rs181316459、rs2664280、rs7310615、rs34536443 | |
| PsA | Hyperthyroidism | 12 | 31.518 | 5.12 | rs9461693、rs115625939 | |
| PsA | Hypothyroidism | 10 | 28.508 | 91.58 | rs9461693、rs115625939、rs181316459、rs11085727 | |
| PsA | FT4 | 7 | 10.658 | 59.20 | rs115174302、rs9461693、rs9265889、rs2523560、rs115625939、rs181316459、rs144651842 | |
| PsA | TSH | 7 | 10.658 | 41.80 | rs115174302、rs9461693、rs9265889、rs2523560、rs115625939、rs181316459、rs144651842 | |
| PsA | GD | 12 | 29.785 | 8.36 | rs115174302、rs9461693 | |
| PsA | HT | 11 | 29.736 | 27.62 | rs9461693、rs115625939、rs181316459 | |
| PV | Hyperthyroidism | 5 | 16.489 | 24.98 | rs28895006 | |
| PV | Hypothyroidism | 6 | 66.265 | 90.10 |  | |
| PV | FT4 | 3 | 16.489 | 4.60 | rs3115628、rs17188113、rs28895006 | |
| PV | TSH | 3 | 16.489 | 99.30 | rs3115628、rs17188113、rs28895006 | |
| PV | GD | 5 | 26.892 | 66.99 | rs28895006 | |
| PV | HT | 4 | 55.862 | 22.86 | rs3115628、rs17188113 | |

R^2^ reflects the proportion of exposure variance explained by the Instrumental variable. PsO, psoriasis. PsA, psoriatic arthritis. PV, psoriasis vulgaris. GD, Graves’ disease. HT, Hashimoto thyroiditis. TSH, Thyroid stimulating hormone. FT4, free thyroxine.
