# Supplementary material for: Causality between thyroid disease and psoriasis: Bidirectional Mendelian randomization analysis
Source: Medicine (Baltimore). 2025 Sep 5;104(36):e43426. doi: 10.1097/MD.0000000000043426 (PMC12419343; doi:10.1097/MD.0000000000043426)
Supplement: Supplementary file 4 [file medi-104-e43426-s004.docx]

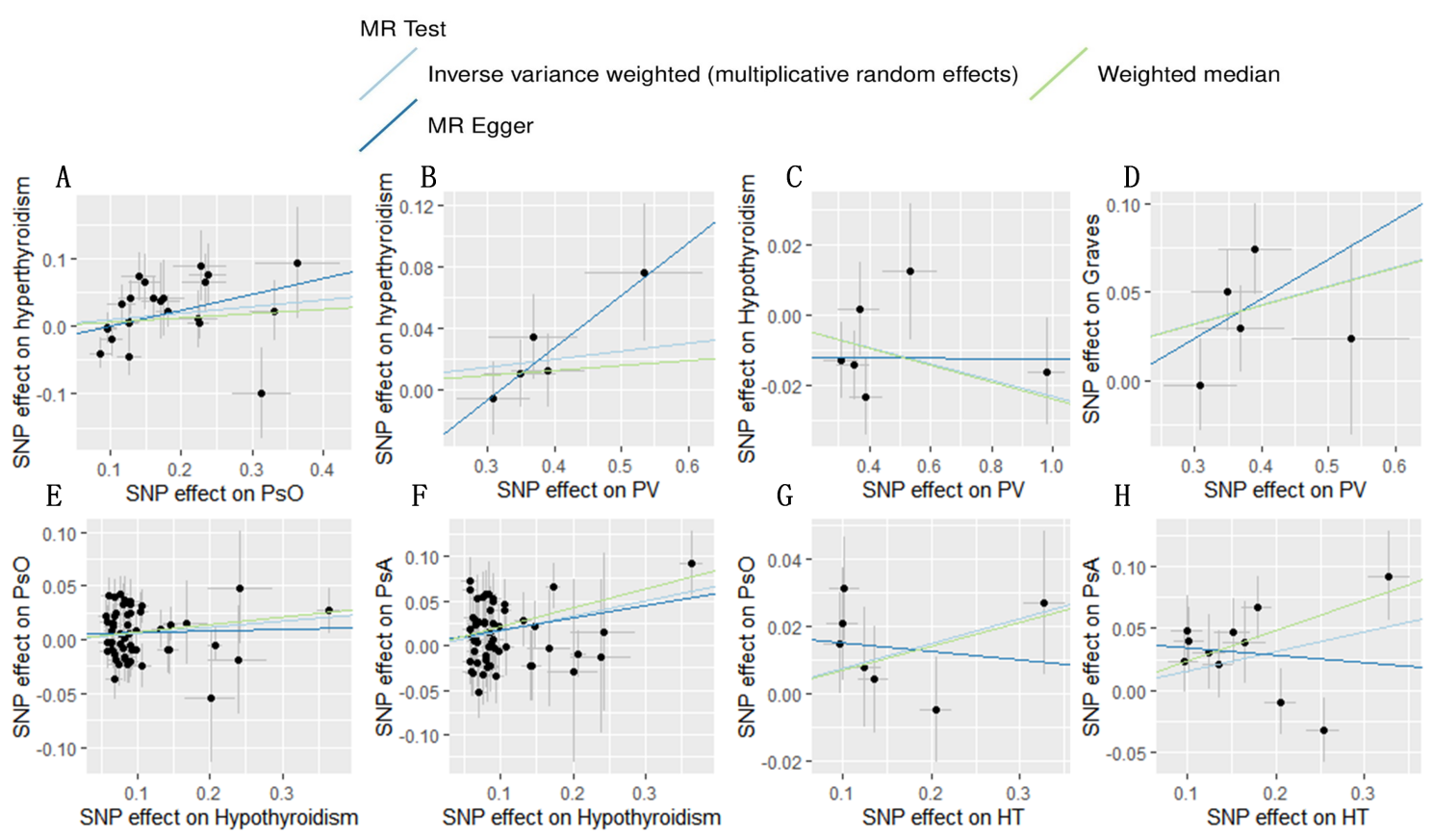


**Supplementary Figure S1.** A scatter plot shows the effects of SNPs. MR stands for Mendelian randomization. The black dot signifies the genetic instrumental variable that is incorporated in the Mendelian randomization analysis. The grey error bar represents the 95% confidence interval for the coefficient of each genetic instrumental variable. Inverse variance weighted (multiplicative randomelian randomization. effects), MR Egger, and weighted median slopes represent results from these regression analyses. (A) A scatter plot shows the effects of SNPs on PsO and Hyperthyroidism. (B) A scatter plot shows the effects of SNPs on PV and Hyperthyroidism. (C) A scatter plot shows the effects of SNPs on PV and Hypothyroidism. (D) A scatter plot shows the effects of SNPs on PV and GD. (E) A scatter plot shows the effects of SNPs on Hypothyroidism and PsO. (F) A scatter plot shows the effects of SNPs on Hypothyroidism and PsA. (G) A scatter plot shows the effects of SNPs on HT and PsO. (H) A scatter plot shows the effects of SNPs on HT and PsA.


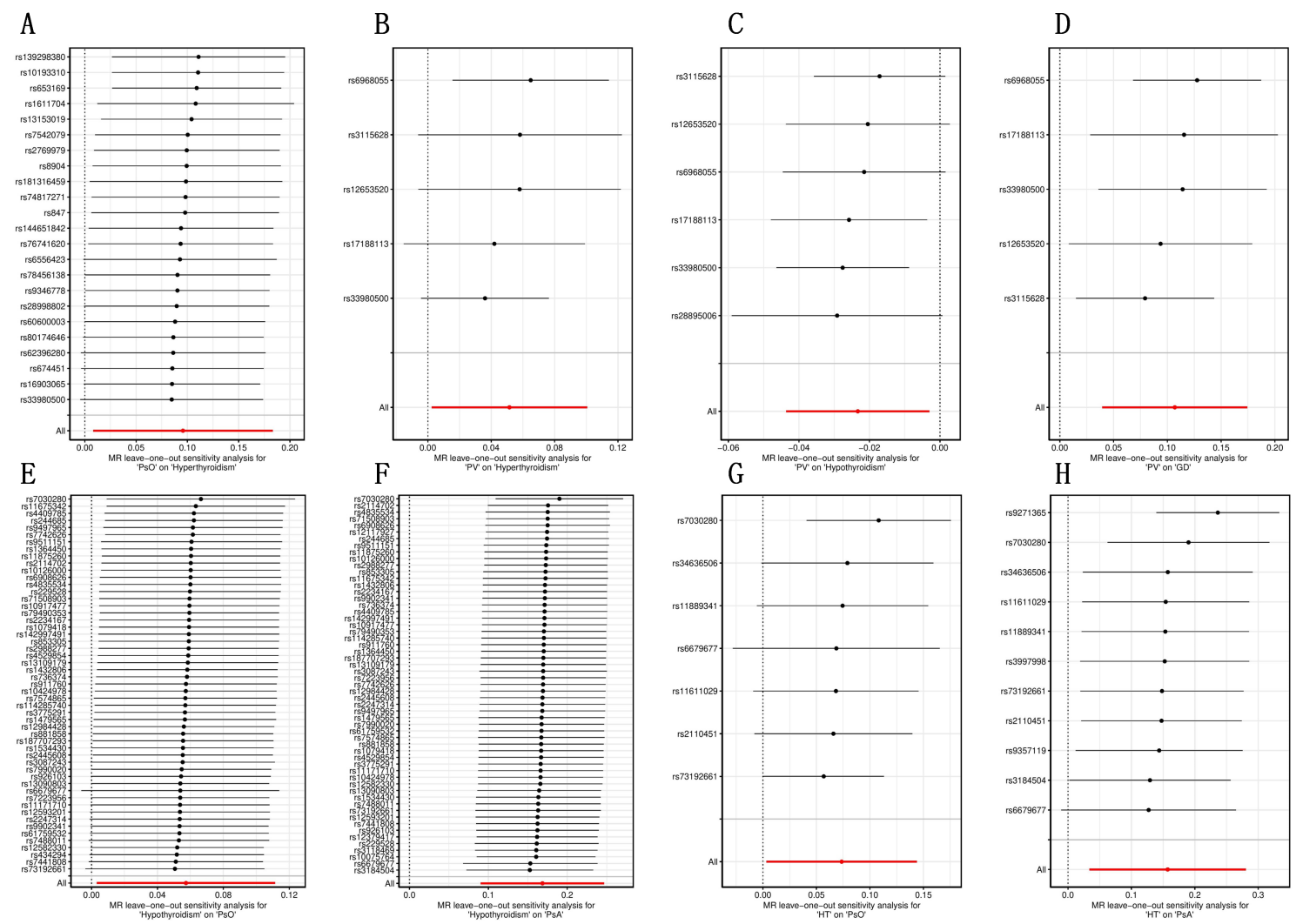


**Supplementary Figure S2.** A leave-one-out analysis of the estimations plot. The black dots represent the effect values of a single SNP, the black lines represent the 95% confidence of the effect values, and the red dots and lines represent the effect values and 95% confidence of the Inverse variance weighted (multiplicative random effects) and MR Egger population, respectively. (A)A leave-one-out analysis of the estimations for PsO and Hyperthyroidism. (B)A leave-one-out analysis of the estimations for PV and Hyperthyroidism. (C)A leave-one-out analysis of the estimations for PV and Hypothyroidism. (D)A leave-one-out analysis of the estimations for PV and GD. (E)A leave-one-out analysis of the estimations for Hypothyroidism and PsO. (F)A leave-one-out analysis of the estimations for Hypothyroidism and PsA. (G)A leave-one-out analysis of the estimations for HT and PsO. (H)A leave-one-out analysis of the estimations for HT and PsA.


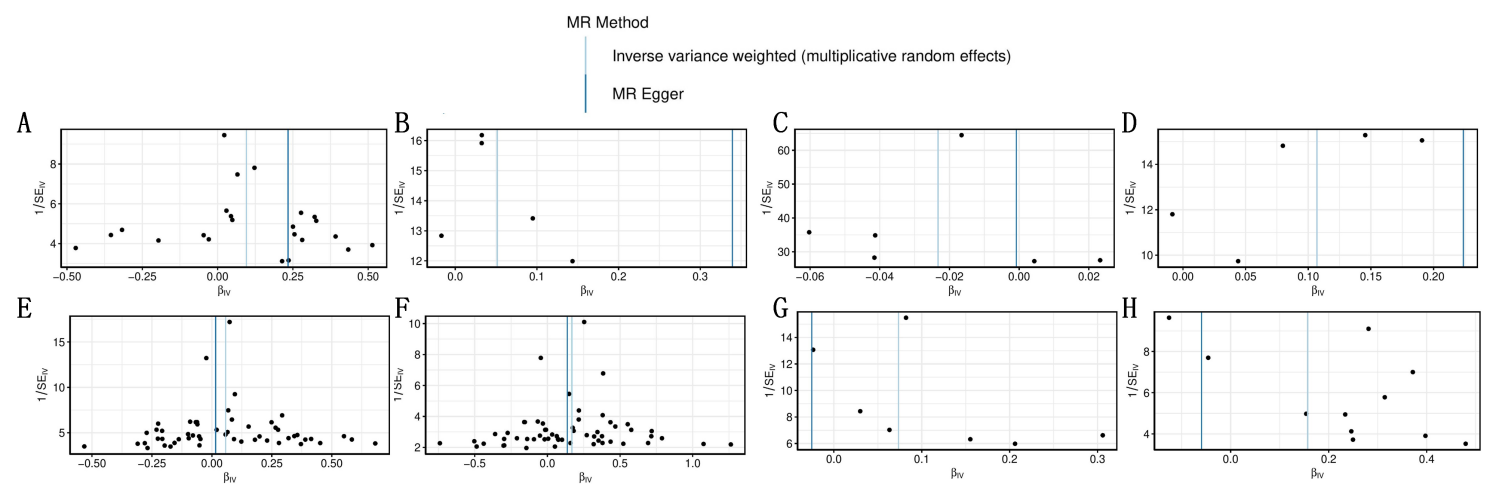


**Supplementary Figure S3.** Funnel plot. MR stands for Mendelian randomization; The black dots represent the effect value of a single SNP and the reciprocal of SE, used to visually observe the degree of dispersion of the results and determine whether there is potential bias or heterogeneity. MR stands for Mendelian randomization. IV, instrumental variable. (A) Funnel plot for the PsO and Hyperthyroidism of SNPs. (B).Funnel plot for the PV and Hyperthyroidism of SNPs. (C).Funnel plot for the PV and Hypothyroidism of SNPs. (D) Funnel plot for the PV and GD of SNPs. (E) Funnel plot for the Hypothyroidism and PsO of SNPs. (F) Funnel plot for the Hypothyroidism and PsA of SNPs. (G) Funnel plot for the HT and PsO of SNPs. (H) Funnel plot for the HT and PsA of SNPs.

**
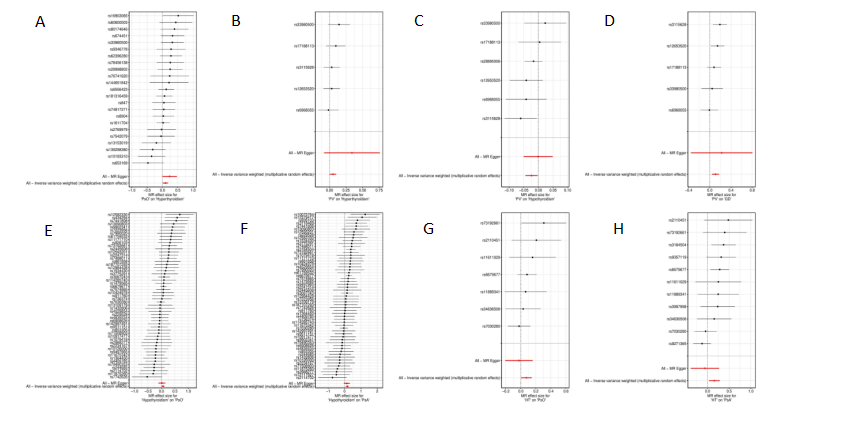
**

**Supplementary Figure S4**. A forest plot. The black dots usually represent the analysis results corresponding to each SNP that has been removed one by one. The black line represents 95% confidence of the effect value, while the red dots and lines represent the overall effect value and 95% confidence of Inverse variance weighted (multiplicative random effects) and MR Egger, respectively. (A)A forest plot of PsO and Hyperthyroidism of SNPs. (B).A forest plot of PV and Hyperthyroidism of SNPs. (C) A forest plot of PV and Hypothyroidism of SNPs. (D) A forest plot of PV and GD of SNPs. (E) A forest plot of Hypothyroidism and PsO of SNPs. (F) A forest plot of Hypothyroidism and PsA of SNPs. (G) A forest plot of HT and PsO of SNPs. (H) Funnel plot for the HT and PsA of SNPs.
