# Supplementary material for: Causality between thyroid disease and psoriasis: Bidirectional Mendelian randomization analysis
Source: Medicine (Baltimore). 2025 Sep 5;104(36):e43426. doi: 10.1097/MD.0000000000043426 (PMC12419343; doi:10.1097/MD.0000000000043426)
Supplement: Supplementary file 6 [file medi-104-e43426-s006.docx]

| exposure | outcome | heterogeneity test | | pleiotropy test | | |
| --- | --- | --- | --- | --- | --- | --- |
|  |  | Q | Q_pval | egger_intercept | se | pval |
| PV | Hypothyroidism | 4.805 | 0.440 | -0.012 | 0.012 | 0.383 |
| PV | FT4 | 1.252 | 0.535 | -0.026 | 0.023 | 0.469 |
| PV | GD | 4.333 | 0.363 | -0.043 | 0.108 | 0.717 |
| PV | HT | 1.500 | 0.682 | 0.001 | 0.022 | 0.968 |
| PV | Hyperthyroidism | 2.515 | 0.642 | -0.107 | 0.079 | 0.269 |
| PV | TSH | 2.151 | 0.341 | 0.022 | 0.024 | 0.528 |
| PsA | Hypothyroidism | 15.278 | 0.084 | -0.008 | 0.018 | 0.665 |
| PsA | FT4 | 11.051 | 0.087 | 0.060 | 0.026 | 0.070 |
| PsA | GD | 19.550 | 0.052 | 0.075 | 0.029 | 0.059 |
| PsA | HT | 7.381 | 0.689 | 0.003 | 0.016 | 0.865 |
| PsA | Hyperthyroidism | 18.907 | 0.063 | 0.072 | 0.028 | 0.068 |
| PsA | TSH | 3.434 | 0.753 | 0.021 | 0.024 | 0.426 |
| PsO | Hypothyroidism | 27.526 | 0.070 | -0.011 | 0.011 | 0.321 |
| PsO | FT4 | 27.480 | 0.122 | 0.003 | 0.007 | 0.639 |
| PsO | GD | 28.910 | 0.224 | -0.020 | 0.018 | 0.272 |
| PsO | HT | 21.277 | 0.504 | 0.013 | 0.013 | 0.327 |
| PsO | Hyperthyroidism | 27.152 | 0.206 | -0.023 | 0.019 | 0.230 |
| PsO | TSH | 21.277 | 0.381 | 0.000 | 0.006 | 0.937 |
| FT4 | PV | 24.553 | 0.219 | -0.048 | 0.038 | 0.216 |
| FT4 | PsA | 23.128 | 0.283 | -0.004 | 0.018 | 0.834 |
| FT4 | PsO | 28.637 | 0.095 | -0.013 | 0.011 | 0.266 |
| GD | PV | 33.431 | 0.042 | -0.024 | 0.050 | 0.629 |
| GD | PsA | 20.839 | 0.234 | 0.022 | 0.023 | 0.358 |
| GD | PsO | 27.832 | 0.065 | 0.002 | 0.015 | 0.909 |
| HT | PV | 10.969 | 0.203 | 0.093 | 0.053 | 0.123 |
| HT | PsA | 16.800 | 0.079 | 0.040 | 0.029 | 0.194 |
| HT | PsO | 5.027 | 0.540 | 0.017 | 0.015 | 0.292 |
| Hyperthyroidism | PV | 8.049 | 0.624 | 0.005 | 0.048 | 0.911 |
| Hyperthyroidism | PsA | 9.656 | 0.290 | 0.042 | 0.023 | 0.104 |
| Hyperthyroidism | PsO | 14.069 | 0.080 | 0.013 | 0.026 | 0.636 |
| Hypothyroidism | PV | 79.247 | 0.058 | 0.023 | 0.020 | 0.248 |
| Hypothyroidism | PsA | 59.217 | 0.359 | 0.004 | 0.009 | 0.702 |
| Hypothyroidism | PsO | 68.506 | 0.062 | 0.005 | 0.006 | 0.470 |
| TSH | PV | 48.632 | 0.164 | 0.009 | 0.029 | 0.756 |
| TSH | PsA | 38.951 | 0.517 | -0.006 | 0.012 | 0.595 |
| TSH | PsO | 31.856 | 0.785 | -0.004 | 0.007 | 0.579 |

Supplementary Table S4 heterogeneity and horizontal pleiotropy test after final MR
